# Vertical Transmission and Discordance of Cytomegalovirus in Twin Pregnancies

**DOI:** 10.3389/fcimb.2021.676988

**Published:** 2021-07-19

**Authors:** Jill Hutton, Paul J. Rowan

**Affiliations:** ^1^ Mednax, Sunshine, FL, United States; ^2^ Autism Research Texas, Houston, TX, United States; ^3^ Division of Management, Policy, and Community Health, The University of Texas-Houston School of Public Health, Houston, TX, United States

**Keywords:** hereditability, CMV, cytomegalovirus, congenital, infection, twin, vertical transmission

## Abstract

**Objective:**

The objectives are to estimate the vertical transmission rate in twins relative to singleton pregnancies, to evaluate whether discordance within twin pairs is rare, and to characterize concordance within monozygotic and dizygotic twin pairs in relation to hereditability.

**Methods:**

We first sought to estimate the vertical transmission rate of congenital CMV infection in twins by gathering cohort-based studies of congenital CMV in which vertical transmission in both singleton and twin pregnancies was reported. This also allowed us to compare singleton and twin infection rates. From the above studies and other large cohorts of congenitally infected infants, the percentage of discordantly infected twin pairs determined whether this is a rare phenomenon. Theorizing discordance is not rare, we then analyzed data from cases with twin outcomes for congenital CMV infection, according to whether the twins were monozygotic or dizygotic, and calculated their corresponding concordance rates to estimate the broad-sense heritability. Lastly, we described other factors that might affect vertical transmission.

**Results:**

From five articles following at-risk pregnancies, the rate of vertical transmission in twin pregnancies is 58.7% (95% CI 43.3-72.3%) whereas in singleton pregnancies it is 31.4% (95% CI: 29.0-34.0%) *p* = 0.0002. Of ten studies of larger cohorts of infants with congenital CMV infection, 21 of 42 twin pairs with at least one twin infected were discordant for congenital CMV (50.0%, 95% CI: 34.4–65.6%) indicating discordance of congenital CMV infection in twin pairs is not rare. Of 28 studies covering 37 twin pairs where at least one twin had congenital CMV, and zygosity was known, eleven of thirteen monozygotic twin pairs (84.6%; 95% CI: 53.7-97.3%) were concordant for CMV infection, and nine of twenty-four dizygotic twin pairs (37.5%; 95% CI: 19.6-59.2%) were concordant for infection giving an estimated hereditability of 94.2%. Within these 37 twin pairs, factors such as primary or recurrent maternal infection, prematurity, growth discordance, and sex are described; however, in many of these cases these factors are unknown.

**Conclusion:**

The rate of vertical transmission of congenital CMV is higher for twins than singletons. Discordance of congenital CMV in twins is not rare and suggests a possible genetic susceptibility to congenital CMV.

## Highlights

- Vertical transmission of congenital CMV infection in twins is higher than that of single pregnancies.
- Discordance of congenital CMV infection in twins is not rare.
- Examining patterns of concordance between monozygotic and dizygotic twins suggests a genetic susceptibility to congenital CMV infection.
- Causes of genetic conditions with poorly understood etiology might include infectious agents.

## Introduction

A congenital infection in pregnancy can discordantly affect twins. This phenomenon is getting increased attention. In a recent study examining concordance for congenital Zika syndrome in twins (Caires-Júnior et al., 2018), both of the two monozygotic (MZ) pairs examined were concordant, but only one of seven dizygotic (DZ) pairs examined was concordant. Since Zika is a relatively new teratogen, concordance data for congenital Zika in MZ and DZ twins are limited to evaluate discordance and support the idea of genetic susceptibility to a congenital infection.

Whereas Zika is a relatively new teratogen with limited data, epidemiological data for cytomegalovirus (CMV, Human betaherpes 5 virus) span several decades. CMV is the most common congenital viral infection in many populations throughout the world, with a prevalence of approximately 0.5% of all births (Hutton, 2018). Like the Zika example above, one twin may become congenitally infected with CMV, while the other twin shows no evidence of infection. This phenomenon spurs many case reports, but the rate of discordantly infected twins is not known, nor whether this occurrence is common or rare. Further, the vertical transmission rate of congenital CMV in twin pregnancies is largely unknown.

The first aim of this paper is to estimate the vertical transmission rate of congenital CMV infection in twin pregnancies and to verify discordance in population-based studies. Using large cohorts of at-risk pregnancies inclusive of twins, we will determine a vertical transmission rate of CMV in twins and compare it to the rate of singleton pregnancies as extracted from the same articles. Further, we aim to look at discordantly infected twin pairs in large cohorts of congenitally infected infants to assess whether CMV discordance is a common occurrence.

The next aim of this article is to better characterize cases of discordance by zygosity. We will gather case studies and series reporting outcomes of congenital CMV infection, according to whether the twins are MZ or DZ. The difference in CMV discordance between MZ twin pairs and DZ twin pairs will be statistically tested, and if different, an estimate of heredity will be given. Other factors affecting possible discordance will also be described.

Finally, the degree of twin discordance between the larger cohort studies and the case studies and series are tested to evaluate whether the twin pair discordance rate in the case studies and case series are generally representative of congenital CMV discordance in twin pairs.

## Materials and Methods

This original research consists of literature searches and is therefore IRB exempt. For the first aim, a literature search of PubMed/Ovid/Medline for “congenital infection + cytomegalovirus + transmission +neonate” was conducted to gather cohort-based studies of vertical transmission of CMV in offspring who were at risk of CMV infection due to maternal CMV infection during pregnancy as given by maternal serology of either seroconversion or detection of IgM. This set of studies was used to estimate the vertical transmission and discordance of CMV in twins. Vertical transmission was calculated separately for singletons and for twins per fetus. Percentages were given with 95% confidence ratios, and their significance of difference between two independent proportions, and relative risk were calculated using VassarStats (Lowry, n.d.). Vertical transmission stratified by gestational age at time of maternal infection in both singletons and twins was also estimated with percentages, 95% confidence ratios, significance of difference, and relative risk. Pending sufficient numbers of studies and participants, a statistical meta-analysis was planned. In order to further evaluate discordance of congenital CMV in twins, an additional set of cohorts of infants with detected congenital CMV was also examined and extracted from this initial literature search. Both sets of cohorts included twin outcomes per fetus and diagnosed congenital CMV in accordance with the National Congenital Cytomegalovirus Disease Registry Collaborating Group (Istas et al., 1995) (further described below). For all twin-pair data in these studies, twin-pair discordance was calculated as the total number of discordant pairs divided by all twin pairs. The first set of cohorts includes twins who are discordant for CMV, and both concordantly positive and concordantly negative for CMV. The second set of cohorts includes twins who are discordant for CMV, and only concordantly positive for CMV. A combined twin discordance of these two sets of studies was then calculated by dividing the total number of discordant twin pairs by the total number of twins excluding the concordantly negative pairs.

The quality of the above articles was scored as per the National Institute of Health Quality Assessment Tool for Observational Cohort and Cross-Sectional Studies. The two authors separately scored each article, and discrepancies were reconciled. Articles of “good” quality or better were included.

To achieve the second aim, a literature search was conducted by searching PubMed/Ovid/Medline for “congenital infection + cytomegalovirus + twin” to gather case series and studies reporting twin outcomes for CMV per fetus that also included whether the twin pairs were MZ or DZ. For a study to be included, proof of congenital CMV infection in each of the twins, at birth, must be clinically evident. Proof could include: 1. polymerase chain reaction (PCR) testing, viral isolation or culture of any of the following: amniotic fluid, urine, serum, dried blood spots, placental swab or secretions; and/or 2. pathology findings such as inclusion bodies or staining methods. Diagnostic tests for CMV could occur from any time in utero to within 3 weeks of birth as per the National Congenital Cytomegalovirus Disease Registry Collaborating Group (Istas et al., 1995).

In some studies, there may be no information on zygosity, and the twin pairs may only be listed by chorions and amnions. Dichorionic-diamniotic (di-di) twins consisting of a female and male are obviously not identical and therefore can be categorized as dizygotic. If a study had such data, it was included; all other di-di twin pair data were not included in this analysis as they cannot be verified to be MZ or DZ.

In addition to lack of classification of zygosity of a twin pair, and lack of proof of infection, other exclusions include postnatally acquired infections, and articles regarding immunodeficient populations. Obtained studies were reviewed to determine whether any two, or more, studies drew upon the same, or overlapping, data sets. If any two (or more) studies drew upon overlapping data sets, the studies would be reviewed to determine which one would be included in this study; the remainder would be excluded.

With the obtained case studies and series, data were abstracted for analysis. Of the MZ twin pairs, some twin pairs are concordantly infected by proof of congenital CMV infection isolated from both individuals; while other sets are discordant with only one infected by proof of congenital CMV infection isolated from only one individual.

For the MZ twin pairs, and separately for the DZ twin pairs, the concordance ratio (CR) was calculated: the number of CMV-concordant twin pairs divided by the total number of both concordant and discordant twin pairs. The dissimilarity in CMV concordance between the two types of zygosity was evaluated with Fisher’s exact test, and odds ratio given using VassarStats (Lowry, n.d.). Next, Falconer’s Estimate was used to determine the “broad-sense” heritability of congenital CMV infection (Falconer’s Estimate: Weber, 2008; Mayhew and Mayre, 2017, pp. 106 - 108). Briefly, Falconer’s Estimate is equal to doubling the difference of CRs between MZ and DZ twins.

We also described, in the cases where data were available, CMV discordance for other factors known to be involved in perinatal disease susceptibility. With small numbers, these examinations are considered exploratory. Factors included: sex (possible for DZ twin pairs; Picone et al., 2005; Dinkar and Singh, 2020); whether the maternal infection was primary or non-primary (Goncé et al., 2012); the gestational age at which the maternal infection occurred (Samedi et al., 2016); prematurity (Shmueli et al., 2017); and fetal growth discordance (Ahlfors et al., 1988). We also examined CMV infection concordance according to whether a MZ twin pair was monochorionic or dichorionic (Ahlfors et al., 1988). While all DZ twins are dichorionic, MZ twins can be monochorionic or dichorionic, with most, 70-74%, being monochorionic (Marceau et al., 2016).

Finally, the degree difference in the proportions of twin discordance between the smaller case studies and series and the larger cohort studies was tested by a chi-square test to verify that the smaller studies do not exaggerate the number of discordant cases.

## Results

This literature search was conducted from October 2019 through June 2020. The literature search for the first aim yielded 462 articles. Of these, the title or abstract of 90 mentioned “twin” or “twins”. Review of these for relevant articles, and the reference lists of relevant articles, yielded the final study set of five that included information regarding CMV infection discordance of twins within cohorts reporting vertical transmission, and five providing information regarding CMV infection discordance of twins within cohorts reporting congenitally infected infants (**Table 1**). None of these studies had duplicate or overlapping samples; however, 3 articles have a twin case or series embedded within the cohort and are also included within the second data set (**Table 2**), specifically articles labeled (Leisnard et al., 2000; Goncé et al., 2012; Lidehäll et al., 2013).

**Table 1:** Vertical transmission of clinically suspected CMV infection in pregnancy in studies making reference to twins.

Study	Number of infants tested (Number of twin pairs)	Number of infants with congenital CMV (Number of individual twins Infected)	Number of twin pairs by concordance pattern		
			+ +	+ -	- -
**Studies of entire newborn cohorts at risk per maternal status**					
Bodéus et al., 2010	537 (13)	250 (18)	8	2	3
Leyder et al., 2016	358 (3)	69 (5)	2	1	0
Picone et al., 2013	241 (3)	60 (3)	0	3	0
Liesnard et al., 2000	210 (2)	55 (1)	0	1	1
Lipitz et al., 1997	65 (2)	22 (0)	0	0	2
Totals, at-risk set	1,411 (23; 3.0%)	456 (27; 5.9%)	10	7	6
**Studies of newborn cohorts with congenital CMV**					
Schmueli et al., 2017	–	508 (24)	6	12	–
Leruez-Ville et al., 2016	–	43 (4)	2	0	–
Yan et al., 2008	–	33 (2)	1	0	–
Lidehäll et al., 2013	–	24 (3)	1	1	–
Goncé et al., 2012	–	19 (3)	1	1	–
Totals, positive set	–	627 (36; 5.7%)	11	14	–
Grand Totals		1,083 (63; 5.8%)	21	21	–

**Table 2:** Congenital CMV concordance, gender, and zygosity in twin pairs reported in CMV concordance case studies and case series; monozygotic twins (top) and dizygotic twins (bottom).

Study	Gender	CMV status	Gender	CMV status
**Monozygotic Twin Pairs**				
Ahlfors et al., 1988	female	+	female	+
Ibid.	female	+	female	+
Ibid.	not reported	+	not reported	+
Bellamy, 1954	not reported	+	not reported	+
McAllister et al., 1964	male	+	male	+
Seguin and Cho, 1988	female	+	Female	–
Nakajima et al., 2015	female	+	female	+
Griesmaier et al., 2010	not reported	+	Not reported	+
Sahiner et al., 2015	Not reported	+	Not reported	+
Kopelman et al., 1972	female	+	Female	+
Baker et al., 1993	male	+	Male	–
Goncé et al., 2012	Not reported	+	Not reported	+
Lidehäll et al., 2013	male	+	male	+
**Dizygotic Twin Pairs**				
Yinon et al., 2006	male	+	male	+
Ibid.	Not reported	+	Not reported	+
Ibid.	Not reported	+	Not reported	+
Ibid.	Not reported	+	Not reported	–
Ahlfors et al., 1988	male	+	Female	–
Ibid.	male	+	female	–
Llorente and Castillo, 2012	male	+	male	–
Ibid.	female	+	female	–
Shearer et al., 1972	male	+	female	–
Morton et al., 1983	male	+	Female	–
Saigal et al., 1982	Not reported	+	Not reported	+
Eachempati and Woods, 1976	male	+	female	+
Samedi et al., 2016	male	+	female	–
Lazzarotto et al., 2003	male	–	female	+
Nigro et al., 1999	male	+	female	+
Kawana et al., 2004	male	+	male	+
Liesnard et al., 2000	Not reported	+	Not reported	–
Agius et al., 1985	male	+	male	+
Hart et al., 1991	male	+	male	+
Kim et al., 2017	male	–	female	+
Lidehäll et al., 2013	male	+	male	–
Gaytant et al., 2003	male	+	male	–
Tomasik et al., 2012	male	–	female	+
Satge et al., 1966	male	+	male	–

The first five articles include 1,411 infants at risk of congenital CMV infection due to a known maternal infection during pregnancy based on maternal serology demonstrating seroconversion or IgM. All five articles attempted to limit their maternal cases to primary infections. Of these infants, 456 (32.3%; 95% CI: 29.9-34.8%) tested positive for congenital CMV infection, including 27 (5.9%) twin individuals.

**Figure 1:**
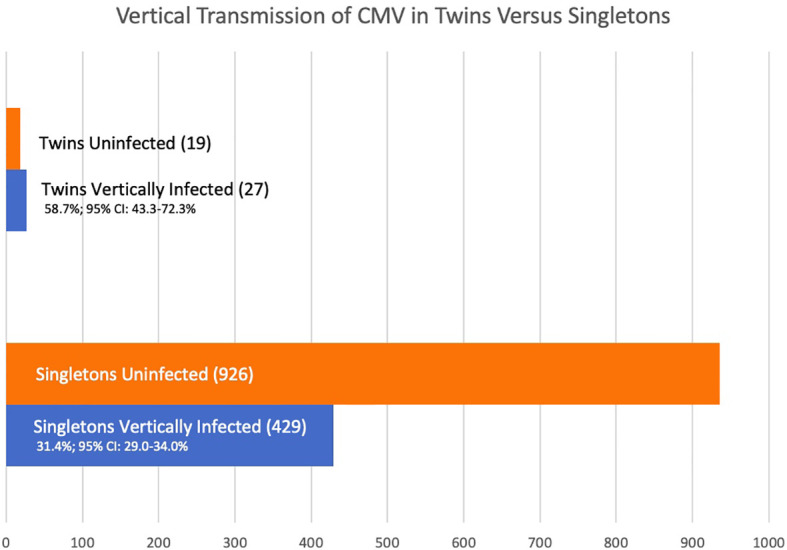
Vertical Transmission of CMV in Twins *Versus* Singletons.

The vertical transmission rate was then examined separately for singletons and for multiples. Of the 1,411 offspring, 1,365 were singletons and there were 23 twin pairs (46 individuals, 3.0%). Of the 1,365 singleton pregnancies, 429 (31.4%; 95% CI: 29.0-34.0%) were vertically infected. Of the 46 individual twins, 27 (58.7%; 95% CI 43.3-72.3%) were vertically infected. So, the rate of vertical congenital transmission was significantly higher in twin pairs than in singletons (**Figure 1**), *p* = 0.0002. The risk ratio is 1.55 (95% CI: 1.1 – 2.1). A statistical meta-analysis was not possible as the number of studies and participants were too small, and one study had zero events.

Stratification by gestational age of maternal infection is limited. Although all five studies stratify by gestational age at time of infection, only one study is clear regarding maternal timing of infection for the twin cases involved. In the Leyder study all maternal infections are listed as occurring at less than 20 weeks’ gestation. In singletons, 64 of 352 (18.2%; 95% CI 14.4 – 22.7%) are vertically infected. Of 6 twin fetuses, 5 (83.3%, 95% CI 36.5 – 99.1%); Fisher’s exact *p* = 0.02, relative risk 3.0 (1.5 – 5.9).

In addition to estimating vertical transmission of congenital CMV in twins relative to singletons, we sought to determine whether discordance of infection was a rare or common event in twins. Looking at discordance of outcome for vertical transmission in these five cohorts of at-risk pregnancies, there are 23 twin pairs, and 7 pairs (30.4%, 95% CI: 14.1–53.0%) were discordant for CMV infection. The denominator of 23 twin pairs includes when both at-risk individuals in the twin pair were negative for CMV. Twin discordance for CMV infection, when at-risk due to maternal infection, is not an uncommon phenomenon.

The above five articles identified infants with congenital CMV as a result of an at-risk pregnancy. To further evaluate discordance in twin pairs, we added in cohorts of newborns with test-proven congenital CMV infections. There were five studies examining outcomes for newborns with congenital CMV (**Table 3**, bottom half). This included 627 infants, with 36 (5.7%) infants congenitally infected from 25 twin pregnancies. Of these twin pairs, 11 (44.0%, 95% CI 25.0 – 64.7%) were concordant, and 14 (56.0%, 95% CI 35.3 – 75.0%) were discordant. Altogether, these 10 studies included a total of 2038 infants inclusive of 96 infants from 48 twin pregnancies; the total number of congenitally infected infants was 1083 with 63 (5.8%) infants from twin pregnancies. From these 10 studies, for all data of twins with at least one twin infected, 21 twin pairs were concordant (both positive) and 21 (50.0%; 95% CI: 34.4–65.6%) were discordant for congenital CMV infection. These various discordance rates indicate that twin discordance is not a rare phenomenon.

**Table 3:** Other possible factors influencing congenital twin CMV concordance.

Factor	Concordant N (%)Zygosity: MZ, DZ	Discordant N (%)Zygosity: MZ, DZ	Fisher’s Exact*
Maternal Infection Primary or Non-Primary			
Primary	5 (14%)	5 (14%)	*p* = 0.99
	1 MZ, 4 DZ	1 MZ, 4 DZ	
Non-Primary	1 (3%)	2 (5%)	
	0 MZ, 1 DZ	0 MZ, 2 DZ	
Unknown	14 (38%)	10 (27%)	
	9 MZ, 4 DZ	1 MZ, 9DZ	
Gestational Stage at Maternal Infection			
Less than 20 weeks’ gestation	5 (14%)	3 (8%)	*p* = n.c.
	0 MZ, 5 DZ	0 MZ, 3 DZ	
Unknown gestational age	15 (41%)	14 (38%)	
	11 MZ, 4 DZ	2 MZ, 12 DZ	
Gestational age at delivery			
Less than 32 weeks	4 (11%)	3 (8%)	*p* = 0.99
	4 MZ, 0 DZ	1 MZ, 2DZ	
33 to 36 weeks	7 (19%)	6 (16%)	
	1 MZ, 6DZ	0 MZ, 6 DZ	
Greater than 36 weeks	3 (8%)	3 (8%)	
	3 MZ, 0 DZ	0 MZ, 3DZ	
Unknown	6 (16%)	5 (14%)	
	3 MZ, 3 DZ	1 MZ, 4 DZ	
Twin growth discordance by body weight			
Greater than 20%	5 (14%)	8 (22%)	*p* = 0.41
	1 MZ, 4 DZ	2 MZ, 6 DZ	
Not greater than 20%	6 (16%)	4 (11%)	
	4 MZ, 2 DZ	0 MZ, 4 DZ	
Unknown	9 (24%)	5 (14%)	
	6 MZ, 3 DZ	0 MZ, 5 DZ	
Chorionic status (MZ twin pairs only)			
Monochorionic	8 (62%)	2 (15%)	*p* = n.c.
Unknown	3 (24%)	0 (0%)	

*Analyses exclude unknown, n.c. = not calculated.

The literature search for the second aim yielded 60 potential studies for review. From this set and their reference lists, as well as reference lists from articles above with embedded twin cases, 28 studies met inclusion criteria, covering 37 twin pairs where at least one twin had congenital CMV. These studies, and the CMV concordance data, are reported in **Table 2**. Given that MZ twins make up approximately one-third of all twins (Marceau et al., 2016), 13 (35%) of 37 twins is a reasonable representation of MZ twins. Two studies had data on both MZ and DZ twin pairs, 9 studies had data on MZ twin pairs only, and 17 studies had data on DZ twin pairs only. The 11 studies reporting MZ twin pairs covered 13 pairs of twins. The 19 studies reporting DZ pairs covered 24 twin pairs. Of the 28 studies, 24 were case reports of solely one twin pair. In 3 articles, the case study or series is written up within a cohort study.

Regarding zygosity, proof varied among the cases and case series. Although many report ultrasounds done for amniocentesis and other purposes, some only labeled the twins as MZ or DZ; specifically, cases labeled Bellamy, McAllister, Sahiner, Lidehäll, Yinon, Liesnard, Agius, and Satge. Ultrasound was specifically used for diagnosis in cases by Ahlfors, Nakajima, Griesmaier, Baker, and Goncé. Pathology was reported by Ahlfors, Seguin, Kopelman, Baker, and Kawana. Ahlfors also used blood typing. Llorente, Saigal, and Kawana followed the twins over one to eight years. The case by Hart used HLA typing, and the case by Gaytant was an *in vitro* pregnancy. Many DZ cases are noted by difference in sex as marked in **Table 2**.

In these studies, concordance of congenital CMV infection was more common in MZ twins than in DZ twins. Eleven of thirteen MZ twin pairs (84.6%; 95% CI: 53.7-97.3%) were concordant for CMV infection. Nine of twenty-four DZ twin pairs (37.5%; 95% CI: 19.6-59.2%) were concordant for infection. The number of concordant MZ twins differs from the number of concordant DZ twins; Fisher’s exact test, *p* = 0.01, OR 9.17 (95% CI: 1.64 – 51.1). The estimate of broad hereditability of congenital CMV infection is 2*(84.6% - 37.5%) = 94.2%.

To examine concordance by sex, information for DZ twin pairs was reviewed. Ten sets were male-female pairs, with eight pairs showing discordance. Of these eight pairs, for five pairs the male was positive for infection, and in three pairs the female was positive. Of the DZ males, nineteen males of twenty-six twin pairs (73%, 95% CI 52.0 – 87.7%) had congenital CMV, whereas six females of the twelve DZ female pairs (50%, 95% CI 22.3 – 77.7%) had congenital CMV infection. Thus, it was more common for a male in a DZ twin pair than a female to be CMV-concordant with an infected twin. This apparent difference was not statistically significant (*p* = 0.16), although this may be due to the small number of cases.

CMV discordance was assessed for other factors known to be involved in perinatal disease susceptibility. Regarding whether maternal infection was primary or secondary, the gestational age at which the infection occurred, and prematurity, there were no discordant patterns (**Table 3**), and as many data were unknown, results could not be calculated for significance. There were no apparent CMV discordances for twin pairs with growth discordance (p-value = 0.41). Of three MZ twins with growth discordance greater than 20%, two were discordant for congenital CMV infection. In one set, the smaller infant had congenital CMV (Seguin and Cho, 1988), and in the other set, the larger infant had congenital CMV (Baker et al., 1993). Of 10 DZ twin pairs with growth discordance, six pairs were discordant for congenital CMV infection, with CMV infection in five of the smaller infants, and one larger infant. Chorionicity of MZ twins was monochorionic in 10 twin pairs and unknown for 3 twin pairs. This is not unexpected with only 13 MZ twin pairs and an estimated 70-74% of all MZ twins being monochorionic (Marceau et al., 2016); however, it does not allow us to evaluate chorionicity.

Finally, the twin-pair CMV discordance rate from the larger cohorts from the first literature search was compared to the discordance rate from the cases and case series from the second literature search. Altogether, these 35 studies included CMV discordance information on 81 twin pairs. Of these, 37 pairs were concordant (both positive) for CMV, 6 pairs were concordant (both negative), and 38 pairs were discordant. Removing the 4 overlapping pairs from the 3 articles labeled (Leisnard et al., 2000; Goncé et al., 2012; Lidehäll et al., 2013), **Table 2** demonstrates 18 concordant (both positive) pairs and 15 discordant pairs; and **Table 1** demonstrates 19 concordant (both positive) pairs and 19 discordant pairs. The discordance portion for these two sets of twins was not statistically different (chi-square statistic = 0.15, *p* = 0.70). This analysis indicates that the discordant cases presented in **Table 2** are not merely over-reported phenomena but are similar in occurrence to what naturally occurs in larger cohorts.

## Discussion

This article estimates that approximately one-third of infants will become infected given a maternal infection with CMV. The vertical transmission rate of CMV for singleton pregnancies is 31.4%, while for twins it is 58.7%. A study by Wang and colleagues (2017) extrapolated the incidence of congenital CMV in singletons (0.7%) and multiples (2.8%) with 70 infants from singleton pregnancies and 3 infants from multiple pregnancies screening positive for CMV out of a population of over 10,000 newborns in a province of China. Here, we better estimate the vertical transmission rate of congenital CMV in twins given maternal infection and show the risk of vertical transmission in twin pregnancies is greater than that of singleton pregnancies. One possible confounding factor is estimated gestational age at time of maternal infection with the vertical rate of transmission rising from 21% in the first trimester, 37% in the second, and 66% in the third trimester (Chatzakis et al., 2020). Our stratification by gestational age of infection is perhaps incomplete as it is limited to a single study, that of Leyder, with the only designation that all maternal infections were prior to 20 weeks’ gestation. In this study, twins had more vertical transmission than singletons. Another indicator that twins have a greater vertical transmission rate is the fact that in the total at-risk infants (upper half of **Table 1**), twins make up 3.0%, but of infants with proven congenital CMV, twins make up 5.9%.

The five cohort articles used to calculate rates of vertical transmission all aimed to identify primary maternal infections, even though use of IgM alone does not completely rule out non-primary infections (Liesnard et al., 2000). The five cohorts all use similar inclusion protocols with regards to maternal infection and congenital infection adding to their homogeneity and reducing detection bias.

Using larger cohorts showing vertical transmission of congenital CMV in infants, and also cohorts describing groups of congenitally infected infants with embedded twin outcomes, many cases of discordance within twin pairs emerge. The aggregate of the 10 studies in **Table 1** demonstrate that the discordance of congenital CMV infection in twins where one twin is infected and the other twin is not infected is not a rare event. Looking at any pair where either one or both infants test positive for congenital CMV, some 50% of the pairs are discordant for infection. These are not exceptional nor rare cases.

If genetics plays a role, then monozygotic twins would show more concordance for infection than dizygotic twins; whereas if this is mere chance, then all should be affected equally. Given the data in **Table 1**, in any twin pair where, one tests positive for CMV, there should be equal portions of concordantly positive and discordant pairs. Looking at the top of **Table 3**, combining MZ and DZ twins, 20 (54%) sets were concordant, and 17 (46%) sets are discordant. This roughly falls in line with a 1:1 ratio. If the sets are evaluated based on zygosity, eleven of thirteen MZ twin pairs (84.6%; 95% CI: 53.7-97.3%) were concordant for CMV infection, whereas nine of twenty-four twin pairs (37.5%; 95% CI: 19.6-59.2%) were concordant for infection. Even given the small numbers, these are statistically different (*p* = 0.01, Fisher’s exact test). These results suggest that genetic susceptibility may be involved. For this reason, an estimate of hereditability is then calculated and estimated at 94%. This estimate of heritability is perhaps overly simplistic, as other factors may also explain different infectious outcomes in twin pairs.

Although preliminary, these data on CMV transmission add to the small but emerging data that congenital infections may follow patterns of hereditability. In the introduction above, a case series of congenital Zika infection showed concordance of infection in two MZ offspring, but only one of seven DZ twins (Caires-Júnior et al., 2018). As further example, a study by Peyron et al. (2003) of clinical congenital toxoplasmosis in twin pairs showed 85% concordance in MZ twin pairs and 44% concordance in DZ twin pairs. All of these examples are rather small in case numbers yet point to a genetic susceptibility.

Hereditability may explain why concordance of congenital CMV infection within MZ twins exceeds that of DZ twins, but there might be other factors to explain this difference. Placentation and blood flow physiology may have some influence on the concordance of congenital CMV infection. All DZ twins are dichorionic, but in MZ twins roughly 70% are monochorionic and 30% are dichorionic (Marceau et al., 2016); therefore, only MZ twins are used to evaluate placentation. Looking at **Table 3**, ten of the MZ pairs are monochorionic, and the chorionicity of three pairs are unknown; hence, we are unable to evaluate chorionicity in this data set. Within the MZ twins in **Table 2**, two sets of mono-di MZ twins are discordant for infection (Seguin and Cho, 1988), including one with twin-twin transfusion syndrome where the recipient/larger twin is infected (Baker et al., 1993). We cannot evaluate chorionicity in this data set; however, the discordance of infection in monochorionic twins leads one to ponder about the epigenetics at play. Twin-twin transfusion syndrome is also noted in two other concordant cases of MZ twins labeled (Griesmaier et al., 2010; Nakajima et al., 2015). In the Nakajima study, the smaller/donor twin was more profoundly affected, while in the Griesmaier study, both twins are affected. In twin-twin transfusion syndrome, the recipient twin might have greater viral exposure or better viral clearance given his/her greater blood flow, while the donor twin might have less viral exposure or worse viral clearance given his/her diminished blood flow. Furthermore, lack of nourishment may worsen the donor twin’s outcome, but these are speculative. Chorions and blood flow may or may not influence congenital infection. To better characterize the influence of hereditability, placentation, and blood flow in regard to congenital CMV, larger studies of at-risk twins with careful placental pathology, and determination of zygosity, would be necessary.

Another difference between MZ and DZ twins is the fact that DZ twins can have different sexes, while MZ twins cannot as they are identical. Sex is not just a descriptive category, but one that also describes a genetic difference. Within our DZ twin set of **Table 2**, there is a possible trend that males have higher rates of congenital infection, but this is not statistically significant. Given that many di-di cases were excluded with the exception of those known to be male-female pairs, this may have skewed the number of DZ twins in favor of male-female pairs (10 of 24 pairs, 42%); however, even with this finding, due to small numbers we cannot fully evaluate the association of sex on vertical transmission of congenital CMV. The Picone study (2005) showed males and females to be equally infected with congenital CMV, but females had worse outcomes. A recent study by Dinkar and Singh (2020) noted more congenital and childhood infections in males than females for CMV and other congenitally transmitted infections as well. There may be a sex difference in susceptibility in male/female DZ twin pairs that deserves further attention and may even help to explain genetic susceptibilities.

Strengths of this study include a search of articles regarding congenital CMV spanning several decades and using evidence-based diagnoses of congenital CMV. Also, as twins have a higher vertical transmission rate of CMV than singletons, larger cohort studies are more likely to include twin cases, thus aiding our subsequent aims. Regarding the first portion of this study, the vertical transmission rate of congenital CMV in twins is based on studies with similar inclusion regarding at-risk pregnancies but limited in the evaluation by timing of infection as described above. Unfortunately, meta-analyses were not applicable to assess heterogeneity and publication bias. Regarding the second portion of the study, the weaknesses include the small numbers of twins identified as MZ and DZ, and the various methods used to diagnose CMV. Older studies relied more on pathology, and viral culture, whereas more recent studies relied on PCR. One difficulty in compiling cases with regard to zygosity is that many articles were excluded as they described the infants in regard to chorions and not zygosity. Many cases listed as dichorionic failed to report sex of each twin in their pairs. Also, cases had to be clear regarding a congenital diagnosis and not a neonatal diagnosis. Another strength of the study is the fact that cases of congenital CMV infection were proven diagnoses, not relying on subjective evaluation or recall. Also, the diagnosis is made either in utero or at the time of birth when the twin environments have little variation as opposed to a diagnosis made after several decades of life with greater variations in child rearing and life events. One other possible weakness relates to the fact that some 80-90% of congenital CMV infections are asymptomatic (Hutton, 2018), and, therefore never diagnosed. This could mean that in regard to the cases and case series only more severe cases are evaluated, as there were some cases with fetal demises and neonatal deaths, specifically by Ahlfors, Ballamy, McAllister, Baker, Shearer, Morton, and Yinon.

A further thought on these patterns of susceptibility to congenital infection is that some diseases considered to be genetic might actually be caused by an infectious agent. This new concept should spur ideas regarding the underlying causes of certain genetically linked disorders. Some disorders have strong hereditability patterns, but no clearly identified genetic basis. Schizophrenia has long been thought to be linked to maternal influenza infection (Boksa, 2008). Autism is also often linked with maternal inflammation (Ornoy et al., 2016); this suggests that both genetic and environmental influences be considered. These may seem tangential but give relevance to the possible long-term outcomes of congenital infections. This current study suggests that infectious origins should not be overlooked as possible causes of disorders that have patterns implicating heritability.

## Data Availability Statement

The original contributions presented in the study are included in the article/supplementary material. Further inquiries can be directed to the corresponding author.

## Author Contributions

We are both accountable for the content of the work. All authors contributed to the article and approved the submitted version.

## Conflict of Interest

Author JH was employed by the company Mednax.

The remaining authors declare that the research was conducted in the absence of any commercial or financial relationships that could be construed as a potential conflict of interest.
